# Law, ethics, and the human right to science: saying what we mean and meaning what we say

**DOI:** 10.3389/fsoc.2025.1448741

**Published:** 2025-03-06

**Authors:** Thérèse Murphy

**Affiliations:** School of Law, Queen's University Belfast, Belfast, United Kingdom

**Keywords:** law, ethics, bioethics, human rights, international human rights law

## Introduction

Interest in the human right to science is steadily increasing, partly due to the interpretive guidance released in 2020 by the Committee on Economic, Social and Cultural Rights, a quasi-judicial UN body. However, how many ethics committees are using or even aware of this guidance or indeed the right itself? Similarly, how many science professionals' organizations know about the 2012 call by the then UN expert on cultural rights, urging them to develop their codes of ethical standards with reference to international human rights law (UN General Assembly, 2012 para 53)? I suspect that professional organizations and ethics committees familiar with the human right to science are few and far between. I believe that needs to change; specifically, these bodies need what I call “human rights literacy.”

Human rights literacy is not about turning these organizations and committees into legal fora. It is about making them more open to, and curious about, the role and limits of international human rights law. This is important for three reasons: first, it acknowledges that human rights are the closest thing we have to a global ethical discourse on values such as freedom, dignity, and welfare; second, it recognizes that this global ethical discourse has a legal form—namely, international human rights law; and third, by engaging with international human rights law in general and the human right to science in particular, ethics committees and science professionals' organizations could activate and amplify these rights. In brief, by giving meaning in practice to both international human rights law in general and the developing human right to science in particular, ethics committees and science professionals' organizations could enhance both their own human rights literacy and that of the broader community.

As a first step toward human rights literacy, this Viewpoint examines the phrase “law, ethics, and rights.” What follows is primarily an opinion piece, grounded in my expertise in international human rights law, my experience as a member of various ethics committees, and my study of the literature on these bodies. I begin by describing a ricochet effect, which I attribute to the unthinking use of the phrase “law, ethics, and rights.” Then, I outline this ricochet effect in four steps, explaining why it is damaging to international human rights law in general and the right to science in particular. I conclude with a simple prescription.

## Law, ethics, and rights: tracking the ricochet

I teach and research international human rights law, and like many of my peers, I make frequent use of the phrase “law, ethics, and rights.” I use it in classrooms, in ethics committee rooms, and in other settings as well. To add precision, I sometimes alter the connector; for example, I refer to the “ethics of rights” or “the morality of law”, “morality and law” or “morality or law” (Heimer, 2010). At other times, I swap a noun for an adjective; for example, I refer to “ethical lawyering”, “ethical judgments” (Smith et al., 2017) or “legal rights.” Relatedly, I often refer to human rights as a “global ethical discourse” and international human rights law as one form of that global ethical discourse (Porsdam Mann et al., 2020).

I use the phrase “law, ethics, and rights” because it conveys something obvious and important: there is—and there ought to be—a relationship between ethics, law, and human rights. Yet I am also increasingly wary of the phrase. I sense that by rolling law, ethics, and rights into one phrase, we risk triggering a ricochet effect that is harmful to international human rights law, including the still-under-development human right to science (Porsdam and Porsdam Mann, 2021). Specifically, when invoked in relation to ethics or law, or even in relation to rights or equality in general, I find that international human rights law is often represented as vapid, lumpen, or somehow offstage (e.g., Moyn, 2019; Posner, 2014). It feels, in brief, less than it is. In particular, its dual nature—as both legal and ethical—gets lost, as does the necessity for ongoing engagement with rights in practice. In what follows, I will explain this ricochet effect in four steps.

### Step 1

“Law” vis-à-vis “ethics” is the first step in my ricochet argument. In my experience of settings charged with ethical deliberation, law—and thus legal knowledge—tends to be upstream or downstream but rarely cascades through the deliberation. In other words, legal knowledge is seen as relevant only as a trigger for the deliberation (e.g., the issue arises at least in part because of a gap or problem with existing law) or as a tool to implement conclusions reached through the deliberation but not as a part of the deliberation. This harms both the deliberation and law and legal knowledge (Cloatre and Pickersgill, 2020). Consider, for example, an approach that views law as a tool to implement conclusions reached via ethical deliberation. This approach assumes that the law's role is to “make it happen”—that the law is one of the forces that will bring the preferred ethical solution to life (Kirkland, 2023). However, as anyone who has studied the relationship between law and social change will tell you, this is a damaging over-simplification (e.g., Galanter, 1974) and sets the law up for failure. It portrays law as “on tap.” It also casts law and lawyers as having little or nothing to contribute to what ought to be. It reduces them to mechanics and technicians rather than recognizing them as individuals who can, do, and should think about problems in normative ways. If the law recedes in these ways, the risk to the still-developing human right to science is considerable. It will be used only in an instrumental way, narrowing its potential and guaranteeing that it is seen as a disappointment when it fails to demonstrate the levels of bindingness and enforceability that we stereotypically associate with legal norms.

### Step 2

Other stereotypes about law, legal knowledge, and lawyers compound the problem. One example is the assumption that the law's timing is hopelessly out of sync with the timing of science and technology. This assumption implies that either lawmakers dawdle, which could stifle innovation, or that they move too soon, which means a new law could end up regulating improbable, potentially never-to-be innovations. Another stereotype is that technology, once invented, has a life of its own—a sort of unstoppable momentum that is beyond regulation or even moral evaluation (Archard, 2023). I want these stereotypes to be challenged to give the right to science a chance to thrive. We need to ask questions such as how much evidence is there for these stereotypes and are some forms of law more future-proof than others (Jasanoff, 2016)? The UK's pioneering Human Fertilization and Embryology Act, originally introduced in 1990, would be an ideal case study on these issues. It foregrounds process rather than seeking to regulate exclusively via rules that might quickly reach their limits amid technological development or unanticipated uses of technology. More generally, case studies should help promote both human rights in general and the human right to science in particular, giving them a chance to overcome the assumption that the law is always and everywhere a laggard when it comes to governing science and technology. The just-mentioned Human Fertilization and Embryology Act is illustrative here: The commitment of its key regulatory body to public participation, which is integral to the human right to science, ensured that the revision of the law to address mitochondrial donation was much less challenging than anticipated. A further illustration comes from AIDS activism, specifically, the ways in which human rights claims helped to transform an initial regulatory approach, which was mired in criminalization, stigma, and discrimination into a surge of investment in R&D for antiretrovirals and major steps forward in terms of the right to access essential medicines

Another stereotype about law is that it is a “big stick.” When this perception takes hold, understandings of law become centered around prohibition and freedom, which brings its own set of problems. For instance, viewing law as a “big stick” damages international human rights law by making it seem like second-rate law—second-rate because it does not have the allegedly all-important enforceability element characteristic of national law (for example, there is no World Court of Human Rights). What gets occluded by the “big stick” stereotype is not only that law's enforceability, or influence, is a complex phenomenon (De Búrca, 2021) but also that international human rights law has a particular or distinctive character as a form of law—that it is both legal and ethical—and this makes it an exceptionally helpful reasoning tool for difficult issues.

For example, specifically regarding the human right to science, there are many underdeveloped but potentially useful reasoning devices related to this right that could assist ethics committees and science professionals' organizations. The UN's 2020 interpretive guidance features some of these devices, including progressive realization and non-retrogression (CESCR, 2020 paras 23–30). The guidance also uses international human rights law's “right-interference-justification” structure, i.e., does the action that is being restricted fall within the remit or scope of a particular right? If so, has the restriction interfered with the right? If it has, can the restriction be justified as, for example, legitimate and proportionate? (De Schutter, 2019). More generally, instruments such as the 2005 UNESCO-led Universal Declaration on Bioethics and Human Rights, the organization's 2021 Recommendation on the Ethics of AI, and its 2017 Recommendation on Science and Scientific Researchers (UNESCO, 2021, 2017, 2005) are under-utilized starting points for building interest in the potential of rights in general and the right to science in particular.

In summary, if by invoking “law and ethics” we end up juxtaposing law with ethics, we risk poor legal literacy—and particularly poor human rights law literacy by overlooking its dual character as both legal and ethical—which could harm the development of the human right to science.

### Step 3

The third step in my argument begins with a question: When we invoke “ethics and rights,” what meaning is ascribed to “rights?” In my experience, when juxtaposed with ethics, there is a risk that rights are seen as synonymous with freedom or liberty. While this is an important aspect of rights and is crucial for protecting scientific freedom (Porsdam Mann et al., 2023) standing alone, it offers a radically narrow and incomplete account. International human rights law encompasses not only freedoms but also entitlements, obligations, and responsibilities. This is evident even from a cursory reading of any international human rights instrument, starting with the 1948 Universal Declaration on Human Rights. It is also reflected in the UNESCO 2017 Recommendation on Science and Scientific Researchers, which includes a section dedicated to the rights and responsibilities of researchers. It is vital that this understanding of rights as both freedoms and responsibilities cascades through ethics committees and science professionals' organizations. This is vital, for example, to ensure that future recipients of EU Horizon Europe funding do not produce results similar to those of a recent survey, which found that the vast majority of respondents were not familiar with the concept of responsible research and innovation (Bührer et al., 2018). Ethics committees, with their long history of limiting scientific freedom to protect the rights of human research subjects, seem ideally placed to reinforce this core understanding of human rights as being about both freedom and responsibility. However, they must remain vigilant to the increasing threats to legitimate forms of scientific freedom posed by both state and non-state actors.

Seeing rights solely as freedoms is also misleading because it obscures what human rights lawyers refer to as “justified interference” (De Schutter, 2019). For example, the human right to science can justifiably be limited in accordance with the standards outlined in the international instrument in which it is found, namely the UN Covenant on Economic, Social and Cultural Rights. Article 4 of the Covenant first states that limitations on the right to science have to be determined by law; second, they must promote “the general welfare in a democratic society;” and third, any restriction must be compatible with the nature of the right restricted. We risk obscuring this vital dimension of the human right to science (CESCR, 2020 paras 21–22) if we view rights solely as freedoms or liberties. Relatedly, the “justified interference” framework used in international human rights seems an excellent tool for ethics committees and science professionals' organizations when advising on difficult issues.

There is another issue. If rights are treated as synonymous with freedoms or liberties, we risk obscuring equality and non-discrimination as part and parcel of international human rights law—whether as rights in themselves or as cross-cutting principles within this field. There are difficult questions to address regarding equality and non-discrimination in a world increasingly marked by inequality (Fredman, 2022). However, these questions are not best addressed by confining rights to freedoms or liberties and separating equality from international human rights law (De Búrca, 2018). Any perception that human rights are solely about freedom and not about equality would also be particularly damaging to efforts that use the human right to science to support women and girls in science and technology (CESCR, 2020 paras 29–33).

### Step 4

The fourth and final step in my argument focuses on international human rights law in relation to ethics. I have found that, in professional or scholarly ethics fora where I introduce myself as a human rights lawyer, I am sometimes questioned by people from other disciplines about the lack of theory in international human rights law. This could simply be a way of marking territory—an expression of the importance of boxing off “the lawyers” before they try to put themselves “on top” and not just “on tap” (Ashcroft, 2010). Nevertheless, I find it unsettling (Murphy, 2018). In particular, prioritizing theoretical underpinnings risks obscuring the dynamic and iterative nature of international human rights law (De Búrca, 2021)—including the ways in which human rights, as a global ethical discourse, are embraced by individuals and groups advocating for positive change. This includes how the human right to science, as a global ethical discourse, is being taken up by scholars, organizations, and citizen scientists who advocate for and amplify its role as ‘an *ensemble* of “scientific rights”' (Besson, 2024, my emphasis)—encompassing a participatory dimension for all of us to contribute to, not just benefit from, science (CESCR, 2020 paras 10–11).

## Conclusion

The following question arises: What now? What is the way out of the tangle created by invoking “law, ethics, and rights?” Relatedly, how can we move toward human rights literacy within ethics committees and science professionals' organizations so that both the right to science and, more broadly, a human rights-based approach to science are institutionalized as part and parcel of “good science?” I suggest we borrow a framing device that has become popular in discussions about the rights of future generations: an empty chair. In this context, the chair symbolizes the need to consider the rights of future generations when making decisions today. It obliges us, if you will, to be good ancestors.

I believe the empty chair could be repurposed in the context of ethics committees and science professionals' organizations, serving as a reminder of international human rights law as a non-obvious but deeply relevant stakeholder. In brief, an empty chair could act as a threshold gesture, helping us to move toward human rights literacy, including the realization of the neglected potential of the human right to science. Keeping this human right at the forefront has long been challenging. Through the simple device of an empty chair, that tradition could be reversed, stereotypes about the relationship between law, ethics, and rights could be challenged, and the practical meaning of the right to science could develop through the day-to-day work of ethics committees and science professionals' organizations. This, in turn, would feed into state reports to the UN Committee on Economic, Social and Cultural Rights, thereby helping to generate consensus on this right.
